# Expression and Function of StAR in Cancerous and Non-Cancerous Human and Mouse Breast Tissues: New Insights into Diagnosis and Treatment of Hormone-Sensitive Breast Cancer

**DOI:** 10.3390/ijms24010758

**Published:** 2023-01-01

**Authors:** Pulak R. Manna, Sabarish Ramachandran, Jangampalli Adi Pradeepkiran, Deborah Molehin, Isabel Castro-Piedras, Kevin Pruitt, Vadivel Ganapathy, P. Hemachandra Reddy

**Affiliations:** 1 Department of Internal Medicine, Texas Tech University Health Sciences Center, School of Medicine, Lubbock, TX 79430, USA; 2 Department of Cell Biology and Biochemistry, Texas Tech University Health Sciences Center, School of Medicine, Lubbock, TX 79430, USA; 3 Department of Immunology and Molecular Microbiology, Texas Tech University Health Sciences Center, School of Medicine, Lubbock, TX 79430, USA; 4 Department of Pharmacology and Neuroscience, Texas Tech University Health Sciences Center, School of Medicine, Lubbock, TX 79430, USA; 5 Neurology, Departments of School of Medicine, Texas Tech University Health Sciences Center, Lubbock, TX 79430, USA; 6 Public Health Department of Graduate School of Biomedical Sciences, Texas Tech University Health Sciences Center, Lubbock, TX 79430, USA; 7 Department of Speech, Language and Hearing Sciences, School Health Professions, Texas Tech University Health Sciences Center, Lubbock, TX 79430, USA; 8 Nutritional Sciences Department, College of Human Sciences, Texas Tech University, Lubbock, TX 79409, USA

**Keywords:** breast cancer, StAR, aromatase, estrogen/E2, hormone-sensitive BC, mouse models of BCs, HDAC inhibitors, BC therapy

## Abstract

Breast cancer (BC) is primarily triggered by estrogens, especially 17β-estradiol (E2), which are synthesized by the aromatase enzyme. While all steroid hormones are derived from cholesterol, the rate-limiting step in steroid biosynthesis is mediated by the steroidogenic acute regulatory (StAR) protein. Herein, we demonstrate that *StAR* mRNA expression was aberrantly high in human hormone-dependent BC (MCF7, MDA-MB-361, and T-47D), modest in hormone-independent triple negative BC (TNBC; MDA-MB-468, BT-549, and MDA-MB-231), and had little to none in non-cancerous mammary epithelial (HMEC, MCF10A, and MCF12F) cells. In contrast, these cell lines showed abundant expression of aromatase (*CYP19A1*) mRNA. Immunofluorescence displayed qualitatively similar patterns of both StAR and aromatase expression in various breast cells. Additionally, three different transgenic (Tg) mouse models of spontaneous breast tumors, i.e., MMTV-Neu, MMTV-HRAS, and MMTV-PyMT, demonstrated markedly higher expression of StAR mRNA/protein in breast tumors than in normal mammary tissue. While breast tumors in these mouse models exhibited higher expression of *ERα*, *ERβ*, and *PR* mRNAs, their levels were undetected in TNBC tumors. Accumulation of E2 in plasma and breast tissues, from MMTV-PyMT and non-cancerous Tg mice, correlated with StAR, but not with aromatase, signifying the importance of StAR in governing E2 biosynthesis in mammary tissue. Treatment with a variety of histone deacetylase inhibitors (HDACIs) in primary cultures of enriched breast tumor epithelial cells, from MMTV-PyMT mice, resulted in suppression of StAR and E2 levels. Importantly, inhibition of StAR, concomitant with E2 synthesis, by various HDACIs, at clinical and preclinical doses, in MCF7 cells, indicated therapeutic relevance of StAR in hormone-dependent BCs. These findings provide insights into the molecular events underlying the differential expression of StAR in human and mouse cancerous and non-cancerous breast cells/tissues, highlighting StAR could serve not only as a novel diagnostic maker but also as a therapeutic target for the most prevalent hormone-sensitive BCs.

## 1. Introduction

The steroidogenic acute regulatory (StAR) protein (also known as StAR-related lipid transfer protein domain 1, STARD1) mediates the rate-limiting step in steroid biosynthesis, i.e., the transport of cholesterol, the substrate of all steroid hormones, from the outer to the inner mitochondrial membrane [1,2]. Regulation of StAR and hence steroid hormone biosynthesis are obligatory events for the functioning of a variety of cholesterol/steroid led physiological, as well as pathophysiological, processes, which involve endocrine, autocrine, and paracrine signaling [2,3,4]. The expression, activation, and extinction of StAR are influenced by a plethora of signaling events that produce both acute and chronic effects on steroidogenesis. There is increasing evidence that tissue-, stimulus-, and species-specific regulation of various steroid hormones is mediated by mechanisms that enhance transcription, translation, or activity of StAR [2,5,6]. It should be noted that whereas gain-of-function in StAR is associated with increased steroid production, loss-of-function in StAR results in a non-functional and inactive protein that severely deteriorates steroidogenesis, involving hormonal imbalance associated with numerous complications and diseases. Consequently, dysregulation of steroid biosynthesis, affecting androgen and/or estrogen/E2 levels, has been linked to the pathogenesis of a number of hormone-dependent malignancies, including breast cancer (BC) [7,8,9].

Hormone-sensitive BC, the most prevalent non-cutaneous cancer subtype in women globally, evolves due to malfunction in the steroidogenic machinery, in which estrogen and its receptors play vital roles in their progression and survival [10,11,12,13]. In accordance with this, the majority of BCs (~80%) express either estrogen receptor (ER+), especially ERα (*ESR1*), progesterone receptor (PR+), and human epidermal growth factor receptor 2/the erythroblastosis oncogene-B2 (HER2/ErbB2+), or all three, in which E2 is critical for maintenance of these luminal subtype BCs [7,9]. Generally, ER+ BC subtypes are also PR+. Conversely, 15–20% BCs that do not express ER, PR, and HER2 are categorized as triple negative BCs (TNBCs) [14,15]. Despite various cancer subtypes, malignant breast tissues express high levels of aromatase (*CYP19A1*), along with large amounts of estrogens, particularly E2, which are synthesized from androgens by the aromatase enzyme [11,16]. It has been reported that E2 levels in stage specific BCs can be 30 times higher than those seen in their normal counterparts and/or in circulation [12,17,18]. As a consequence, aromatase inhibitors (AIs) have been frequently used to treat and/or prevent BCs in post-menopausal women. Regardless of the effectiveness of endocrine therapy, AIs diminish whole body estrogens and display adverse side effects, including endocrine resistance that is the leading cause of cancer mortality [8,18,19,20]. Noteworthy, however, studies have shown robust expression of aromatase not only in various BC subtypes, but also in non-cancerous breast epithelial cells [21,22,23], warranting improved understanding for diagnosis and therapy for this aggressive disease.

In an earlier report, we demonstrated higher expression of StAR protein in hormone-dependent BCs, in comparison to its moderate level in TNBCs and their non-cancerous counterparts; with no apparent differences in aromatase expression among these three groups [21]. Additionally, amplification of the *StAR* gene, but not other steroidogenic enzyme genes, including aromatase, correlated with poor survival of patients afflicted with luminal subtype BCs [7,9]. We also observed that a number of histone deacetylase inhibitors (HDACIs) was capable of suppressing both StAR and E2 levels in MCF7 cells [21]. A central question is whether StAR could be considered as a diagnostic marker and whether it could serve as a drug target for treatment of hormone-sensitive BC. Noteworthy, dysregulation of HDACs is a primary event in tumorigenesis, since these epigenetic enzymes regulate diverse cellular processes, including cell signaling, chromatin remodeling, and genomic stability through the dynamic process of deacetylation of histone and non-histone proteins [24,25]. Subsequently, HDACIs represent a novel category of drugs for the prevention and treatment of a variety of non-malignant and malignant diseases [25]. Inhibition of HDACs results in acetylation of numerous histone and non-histone substrates, including tumor suppressor proteins and oncogenes [9,26,27]. Utilizing a variety of approaches, our present data provide evidence that StAR is differentially expressed in cancerous and non-cancerous human and mouse breast cells/tissues, underlining the potential of this cholesterol transporter as a novel diagnostic marker for BCs. In addition, we demonstrate suppression of StAR expression, along with E2 biosynthesis, by a variety of HDACIs, not only in hormone-sensitive human MCF7 cells, but also in primary cultures of breast tumor epithelial cells, highlighting therapeutic relevance of StAR as a drug target for treatment of ER+/PR+ BCs. These studies provide mechanistic insights exemplifying the regulatory events by which StAR driven E2 biosynthesis promotes breast tumorigenesis, which opens up a new avenue in BC research.

## 2. Results

### 2.1. Expression of StAR and CYP19A1 mRNA and Protein Levels in Cancerous and Non-Cancerous Human Breast Cell Lines

**Figure 1:**
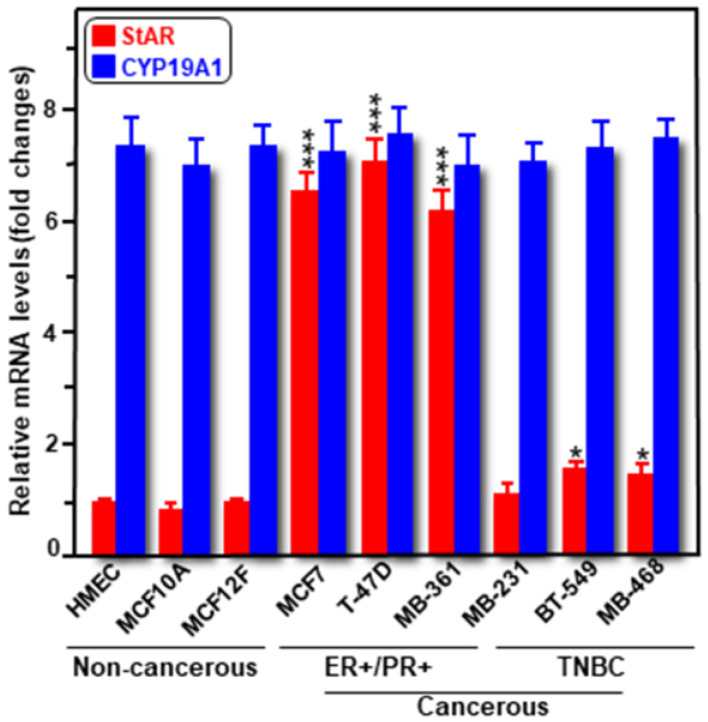
Relative expression of *StAR* and *CYP19A1* mRNAs in human non-cancerous (HMEC, MCF10A, and MCF12F), and cancerous hormone-dependent (MCF7, MDA-MB-361, and T-47D), and hormone-independent (MDA-MB-468, MDA-MB-231, and BT-549) breast cell lines. These cells were cultured with appropriate media and harvested at ~80% confluence, as described under *Materials and methods*. Cells were processed for RNA extraction using PureLink RNA mini kit, and *StAR* and *CYP19A1* mRNA levels were determined by RT-qPCR and reported as fold changes over non-cancerous cells (considered as 1). Results represent the mean ± SE of three to four independent experiments. *, *p* < 0.05; ***, *p* < 0.001 vs. non-cancerous cells.

Expression of both *StAR* and *CYP19A1* mRNAs was determined in a variety of human cancerous ER+/PR+ (MCF7, T47D, and MB-361) and TNBC (MB-231, BT-549, and MB-468), and non-cancerous mammary epithelial (HMEC, MCF10A, and MCF12F) cells. As determined by RT-qPCR, expression of *StAR* mRNA was markedly high (*p* < 0.001) in these three different hormone-dependent BC cell lines compared to the levels seen in either TNBCs or non-cancerous mammary epithelial cells (Figure 1). Specifically, *StAR* mRNA levels were between 6 and 9-fold, and ~1.7-fold, higher in ER+/PR+ and TNBC cells, respectively, over non-cancerous mammary epithelial cells. On the other hand, the expression of *CYP19A1* mRNA was robust, but similar in various cancerous and non-cancerous breast cell lines [22,23]. E2 levels in media correlated with StAR, but not with aromatase, expression in these cells [21]. These data, consistent with our previous findings on both StAR and aromatase protein levels in various human malignant and non-malignant breast cells [21], indicate that StAR expression is abundant in hormone-dependent BC cells, modest in hormone-independent subtypes, with little to none in normal mammary epithelial cells.

**Figure 2:**
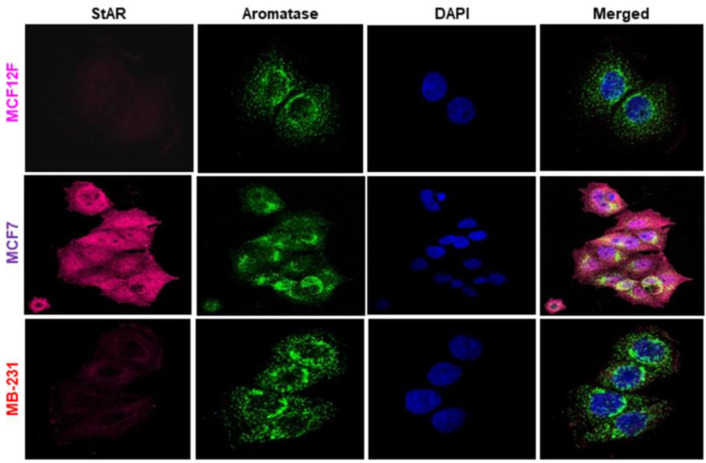
Immunofluorescence analyses of StAR and aromatase in MCF12F, MCF7, and MDA-MB-231 cells, which were grown in 12 mm coverslips placed in 6-well plates to 75–80% confluence. Cells were then processed for immunostaining following procedures described under *Materials and methods*. Images were taken using a Nikon T1-E confocal microscope with a 60X objective. Representative immunofluorescence images (N = 3) illustrate relative expression of StAR, aromatase, DAPI, and their merged groups. Images were taken with the same laser power and gain and represent a maximum projection intensity derived from a Z-stack.

Immunofluorescence analyses were performed to determine expression levels of both of StAR and aromatase proteins, utilizing cancerous MCF7 and MB-231, and non-cancerous MCF12F cells. The results show that expression of the StAR protein is noticeably high in MCF7, modest in MB-231, and nearly undetectable in MCF12F cells (Figure 2). Under similar experimental conditions, robust expression of aromatase protein was observed in all three cell types and there were no significant differences among those categories. These results corroborate data presented in Figure 1 and demonstrate markedly higher expression of StAR protein in hormone-sensitive BC cells compared to hormone-negative MB-231 (TNBC) and normal mammary epithelial MCF12F cells [21]. In contrast, aromatase expression is abundant and invariable in both cancerous and non-cancerous breast cells. These findings imply that StAR plays an important role in the progression of hormone-sensitive BCs.

### 2.2. Differential Expression of StAR, ERα, ERβ, and PR mRNAs, and E2 Levels in Transgenic Mouse Models of Breast Tumors

**Figure 3:**
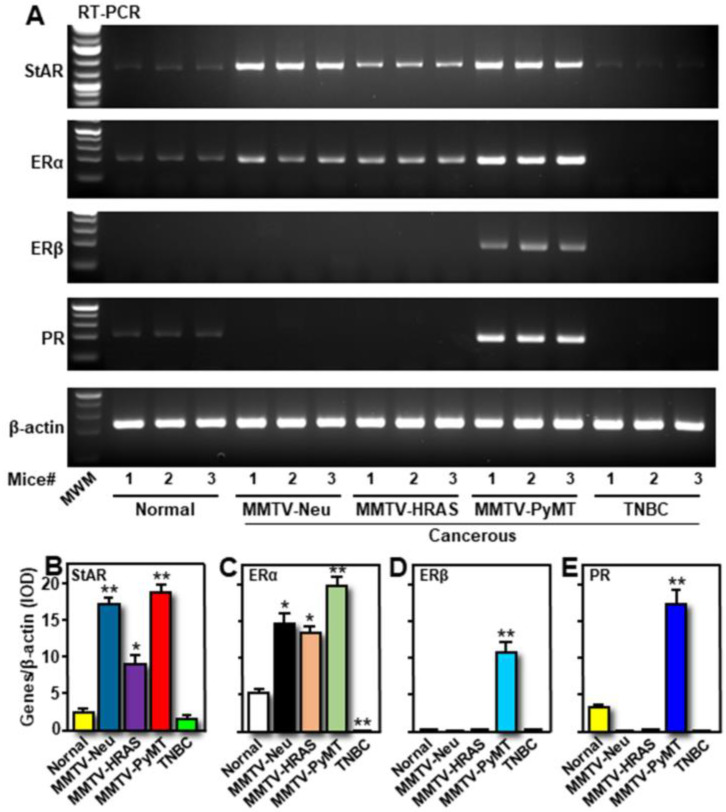
Expression of *StAR, ERα, ERβ,* and *PR* mRNAs in Tg mice breast tumors from MMTV-Neu, MMTV-HRAS, MMTV-PyMT, and TNBC, and in normal mammary tissue. Both non-cancerous and cancerous breast tissues were subjected to total RNA extraction using either TRIzol reagent or PureLink RNA mini kit, and mRNA levels were determined by semi-quantitative RT-PCR (**A**), as described under *Materials and methods*. Representative gel images illustrate expression of *StAR, ERα, ERβ,* and *PR* mRNAs in different mouse groups (three independent mice # 1, 2, and 3 in each category). Integrated optical density (IOD) values for *StAR* (**B**), *ERα* (**C**), *ERβ* (**D**), and *PR* (**E**), in each band, were quantified and normalized with the corresponding β-actin bands, and combined data are presented (N = 3). *, *p* < 0.05; **, *p* < 0.01 vs. Normal. MWM, molecular weight marker.

The hypothesis that hormone-sensitive BCs express aberrantly high expression of StAR, along with increased E2 accumulation, to promote tumorigenesis, was evaluated. Three different Tg mouse models of spontaneously occurring breast tumors utilized were: MMTV-Neu, MMTV-HRAS, and MMTV-PyMT, in addition to one TNBC mouse model and similar genetic background wild-type mice [28,29]. As determined by semi-quantitative RT-PCR, expression of *StAR* mRNA was evidently high in breast tumors induced by *Neu*, *HRAS*, and *PyMT* oncogenes, when compared with either TNBC or normal mouse mammary tissue (Figure 3A,B). MMTV-HRAS-driven breast tumors showed comparatively lower expression of *StAR* mRNA. While breast tumors in MMTV-PyMT mice displayed higher expression of *ERα*, *ERβ,* and *PR* mRNAs, MMTV-Neu and MMTV-HRAS mice breast tumors lacked *ERβ* and *PR* gene expression (Figure 3A,C–E). These results indicate that MMTV-PyMT breast tumors are ER+/PR+, in contrast to ER-negative in a previous study [30]. *StAR*, *ERα*, *ERβ,* and *PR* mRNA levels were undetectable in breast tumors obtained from TNBC mice (Figure 3A–E), confirming that these tumors are hormone-independent. Relatively low levels of *StAR*, *ERα*, and *PR* mRNAs in normal mouse mammary tissue indicate their requirements for normal breast development and function.

**Figure 4:**
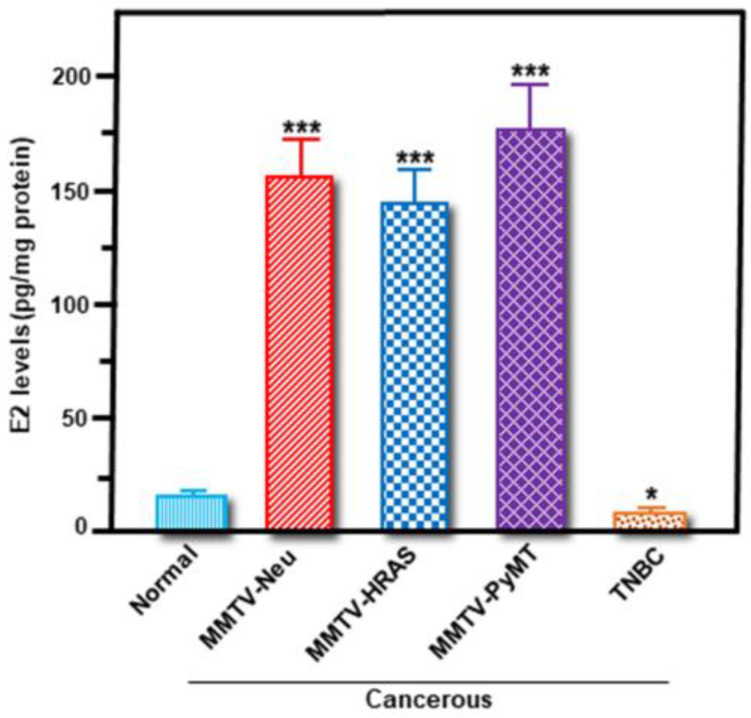
Determination of E2 levels in breast tissues from wild-type mice and four different Tg mouse models of breast tumors. Non-cancerous mammary tissue and cancerous breast tumor tissues collected from wild-type normal, MMTV-Neu, MMTV-HRAS, MMTV-PyMT, and TNBC mice (three different mice in each category) were homogenized in RIPA lysis buffer, and cell lysates from different groups were extracted with dichloromethane as described under *Materials and methods*. Levels of E2 in extracted breast tissue lysates were determined by ELISA, and presented as pg/mg protein. Data represent the mean ± SE of three different mice in each group. *, *p* < 0.05; ***, *p* < 0.001 vs. Normal.

To better understand the significance of these hormone receptor expression levels in cancerous and non-cancerous breast tissues, accumulation of E2 was next determined. It can be seen from Figure 4 that E2 levels were between 5.7 and 9.6-fold higher in breast tumor tissue lysates pertaining to MMTV-Neu, MMTV-HRAS, and MMTV-PyMT mice, when compared with normal mammary tissue. Conversely, E2 synthesis was found to be attenuated (*p* < 0.05) in TNBC breast tumors (Figure 4). The results demonstrating higher *StAR* mRNA and E2 synthesis in three Tg mouse models of hormone-sensitive breast tumors reflected qualitatively similar patterns in human BC cell lines, representing a close correlation between StAR expression and E2 accumulation, along with their association to ER+/PR+ BCs.

### 2.3. Functional Relevance of StAR and Aromatase Expression, and Their Correlation to E2 Accumulation, in Breast Tumor Tissue and Plasma of MMTV-PyMT Mice

**Figure 5:**
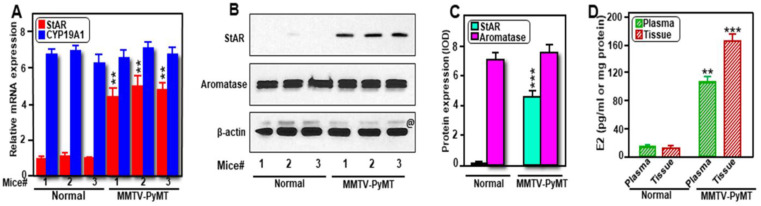
Determination of StAR and aromatase, at mRNA and protein levels, in breast tissues; and E2 levels in plasma and breast tissues from normal and MMTV-PyMT mice. Various breast tissues (3 mice in each group) were subjected to either RNA extraction or whole cell protein preparation, as described under *Materials and methods*. *StAR* and *CYP19A1* mRNA levels were determined by RT-qPCR and combined data are presented (**A**). Representative immunoblots show expression of StAR and aromatase in three independent mice from each category using 70 μg of total protein (**B**). β-actin expression was assessed as a loading control. IOD values for StAR and aromatase, in each band, were quantified and normalized with the corresponding β-actin bands, and combined data are presented (**C**). Both breast tumor tissue lysates and plasma from each mouse were extracted with dichloromethane, and E2 levels were determined by ELISA and presented as either pg/mL (plasma) or pg/mg protein (tissue) (**D**). Results represent the mean ± SE of three independent mice. **, *p* < 0.01; ***, *p* < 0.001 vs. Normal. @, non-specific band.

Since breast tumors in MMTV-PyMT Tg mice were identified as ER+/PR+, we determined expression of both StAR and aromatase levels, and their correlation to E2 biosynthesis. As determined by RT-qPCR (Figure 5A), expression of *StAR* mRNA was 4.6 ± 1.7-fold higher in breast tumors of MMTV-PyMT mice, when compared with normal mouse mammary tissues. *CYP19A1* mRNA levels in both MMTV-PyMT-driven breast tumors and normal mammary tissue were substantially high with no apparent differences between the two groups. Whereas expression of the StAR protein was little to none in normal mammary tissue, its level was markedly high (*p* < 0.001) in breast tumors of MMTV-PyMT mice (Figure 5B,C). In contrast, strikingly higher expression of the aromatase protein (appeared as doublets; [21]), was similar in these cancerous and non-cancerous breast tissues. E2 levels in plasma and breast tumor tissue extracts were 5.2 ± 2.3 and 9.7 ± 4.6-fold higher, respectively, in MMTV-PyMT mice, when compared these values with respective non-cancerous categories (Figure 5D), reinforcing higher levels of both intra-tumoral and circulating E2 levels in human hormone-sensitive BCs [12,17]. These findings suggest that aberrant high expression of StAR in breast tumors of MMTV-PyMT mice resulted in abundant delivery of cholesterol to the mitochondrial membrane, facilitating increased availability of androgen precursors for E2 biosynthesis in promoting breast tumorigenesis.

### 2.4. Inhibition of a Variety of HDACs on StAR and E2 Levels in Primary Cultures of Enriched Breast Tumor Epithelial Cells from MMTV-PyMT Mice

**Figure 6:**
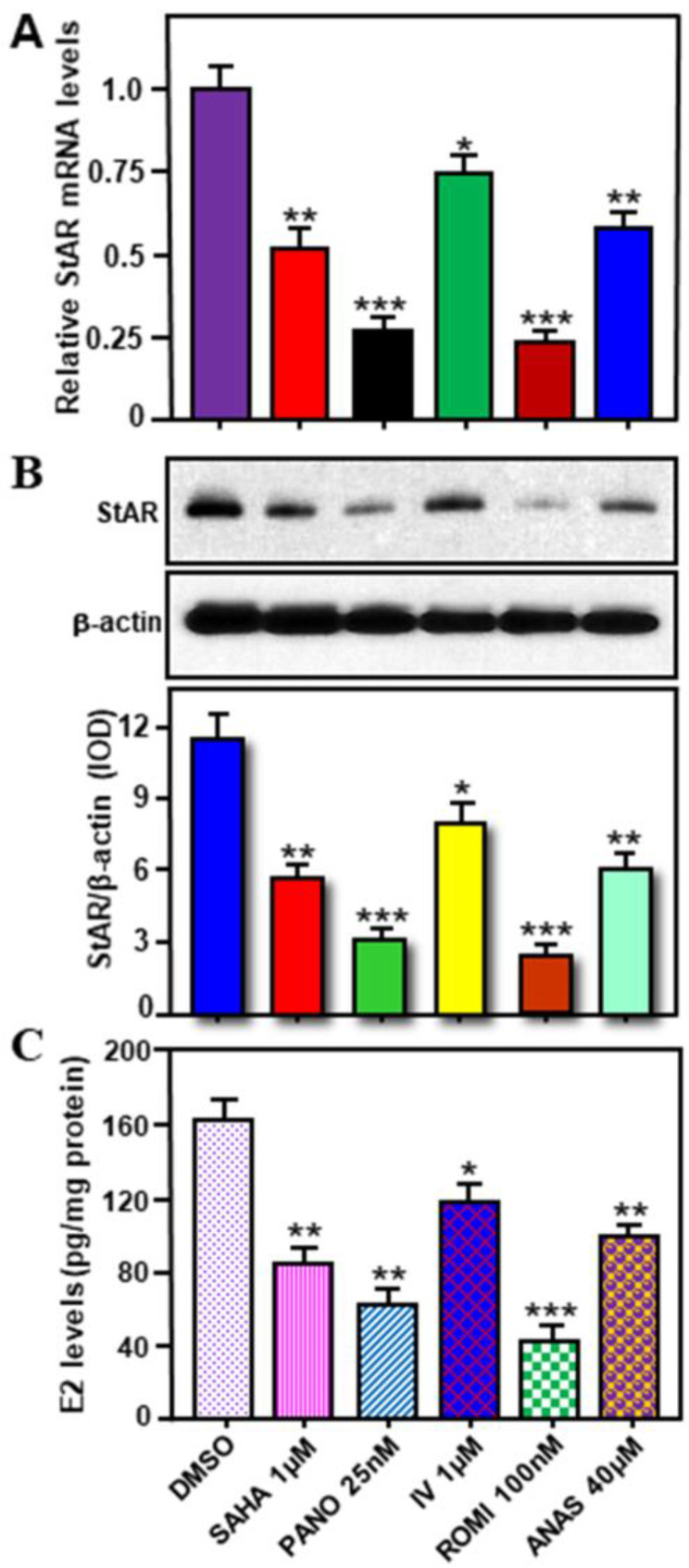
Assessment of StAR expression and E2 accumulation in primary cultures of breast tumor epithelial cells from MMTV-PyMT mice, and their alterations in response to HDACIs. These cells were treated without (DMSO) or with SAHA (1μM), PANO (10 nM), inhibitor IV (IV; 1μM), Romidepsin (ROMI; 1μM), and Anastrozole (ANAS, 40μM), for 24 h. After treatments, cells were subjected to total RNA extraction for determining *StAR* mRNA expression by RT-qPCR (**A**). Cells were also processed for whole cell extract preparation, and representative immunoblots show expression of StAR protein using 60–70 μg of total protein (**B**). β-actin expression was assessed as a loading control. IOD values for StAR and aromatase, in each band, were quantified and normalized with the corresponding β-actin bands, and presented (**B**, bottom panel). E2 levels in media were determined by ELISA and presented as pg/mg protein (**C**). Data represent the mean ± SE of three independent experiments. *, *p* < 0.05; **, *p* < 0.01; ***, *p* < 0.001 vs. DMSO.

We previously observed a link between inhibition of HDACs and StAR/E2 suppression in hormone-sensitive MCF7 cells [21]. To gain more insights into the impact of HDACIs, primary cultures of enriched breast tumor epithelial cells isolated from MMTV-PyMT mice were treated without (DMSO) or with various HDACIs, i.e., SAHA (1μM), Panobinostat (PANO; 10 nM), inhibitor IV (IV; 1μM), Romidepsin (ROMI; 1μM), for 24 h [21,22]. Cells were also treated with Anastrozole (ANAS, 40μM), an AI that has been frequently used for the treatment of BCs in post-menopausal women. As depicted in Figure 6A, higher expression of *StAR* mRNA, in untreated (DMSO) cells, was decreased to varied levels in response to SAHA (49 ± 5.6%), PANO (67 ± 7.3%), and ROMI (72 ± 9.6%). Inhibitor IV, moderately but consistently (28 ± 3.3%), affected *StAR* mRNA expression. Consistent with changes in *StAR* mRNA levels, these HDACIs were also capable of diminishing both StAR protein and E2 biosynthesis to diverse levels (Figure 6B,C). ANAS also reduced (*p* < 0.01) StAR and E2 levels in primary cultures of breast tumor epithelial cells. These results demonstrate that HDACIs, by affecting StAR expression, repress E2 biosynthesis in primary cultures of ER+/PR+ breast tumor epithelial cells.

### 2.5. Effects of Various HDACIs on StAR Expression and E2 Biosynthesis in Hormone-Sensitive MCF7 Cells

**Figure 7:**
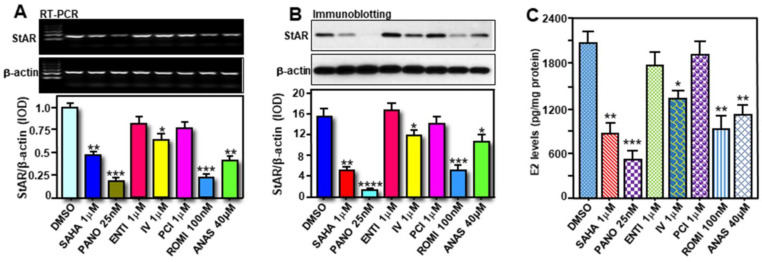
Effects of a variety of HDACIs on StAR expression and E2 synthesis in MCF7 cells. These cells were treated without (DMSO) or with SAHA (1μM), PANO (25 nM), Entinostat (ENTI, 1μM), IV (1 μM), PCI-34051 (PCI, 1 μM), ROMI (100 nM), and ANAS (40 μM), for 24 h. After treatments, cells were processed for total RNA extraction with PureLink RNA mini kit, and *StAR* mRNA expression was determined by a semi-quantitative RT-PCR (**A**). Representative gel images illustrate expression of *StAR* and *β-actin* mRNA levels in different treatment groups. Cells were also homogenized in RIPA lysis buffer and subjected to whole cell extract preparation, and representative immunoblots illustrate StAR protein expression using 50 μg of total protein (**B**). β-actin expression was assessed as a loading control. IOD values for *StAR* mRNA and StAR protein, in each band, were quantified and normalized with the corresponding β-actin bands, and presented (**A,B**; bottom panels). E2 levels in media were determined by ELISA and presented as pg/mg protein (**C**). Data represent the mean ± SE of three to four independent experiments. *, *p* < 0.05; **, *p* < 0.01; ***, *p* < 0.001; ****, *p* < 0.0001 vs. DMSO.

To obtain molecular insights between HDAC inhibition and StAR/E2 regulation, human hormone-sensitive MCF7 cells were treated without (DMSO) or with SAHA (1 μM), PANO (25 nM), Entinostat (ENTI, 1 μM), IV (1 μM), PCI-34051 (PCI, 1 μM), and ROMI (100 nM), at therapeutically relevant doses, for 24 h [21]. Cells were also treated with ANAS (40 μM). The results demonstrate that SAHA, PANO, and ROMI resulted in noticeable decreases (*p* < 0.01) in *StAR* mRNA (Figure 7A), StAR protein (Figure 7B), and E2 biosynthesis (Figure 7C), when compared with respective untreated controls. PANO inhibited StAR and E2 levels between 73 and 90%. SAHA, PANO, and IV moderately inhibited aromatase levels [11,31]. Whereas both ENTI and PCI displayed no apparent effects on StAR expression and E2 synthesis, IV and ANAS inhibited StAR and E2 to varied levels (Figure 7). Inhibition of StAR expression, concomitant with E2 biosynthesis, by various HDACIs, in both human and mouse hormone-sensitive BC cells, underscores the therapeutic relevance of StAR selective for luminal subtype BCs.

## 3. Discussion

BC is a multifactorial condition, and it is influenced by a variety of events, including hormonal, genetic, and reproductive factors. Regardless of diverse events in progression and survival of BCs, an overwhelming amount of evidence indicates that long-term exposure of breast tissue to E2 is a key event in developing tumorigenesis. Whereas aromatization of androgens to estrogens/E2 is achieved by aromatase, its expression is surprisingly high in both non-diseased and diseased breast tissues, indicating that the aromatase-catalyzed event may not be the rate-limiting step in E2 production in mammary tissue [21,22,23]. Cholesterol is the precursor of all steroid hormones, and regulation of steroid biosynthesis is primarily mediated by the StAR protein. We were the first to report that cholesterol transporter, StAR, is abundantly expressed, along with increased E2 levels, in ER+/PR+ BC cells, in comparison to their non-cancerous counterparts [21]. In support of these findings, studies have demonstrated that cholesterol and its metabolites, especially 27-hydroxycholesterol, play crucial roles in the pathophysiology of luminal BC subtypes [32,33,34,35]. Nevertheless, dysregulation of the steroidogenic machinery, involving androgen and E2 biosynthesis, has been linked to BC pathogenesis [10,36,37]. Our present data extend these observations by elucidating the molecular events by which StAR drives hormone-dependent breast tumorigenesis. Specifically, the differential expression of StAR, but not aromatase, in human and mouse cancerous and non-cancerous breast cells/tissues, designates this cholesterol transporter as a novel diagnostic/prognostic marker. Besides, inhibition of StAR, with the corresponding decrease in E2 biosynthesis, by a variety of HDACIs, not only in primary cultures of enriched ER+/PR+ mouse breast tumor epithelial cells, but also in MCF7 cells, signifies the potential of StAR as a drug target for treatment of hormone-sensitive BCs.

The occurrence of genetic abnormality, involving gene amplifications and/or oncogene activation, is a fundamental event in cancer progression and survival. There is increasing evidence that intra-tumoral E2 buildup in malignant breast tissue is due to enhanced expression and activity of aromatase [11,17,31,37,38]. In keeping with this, AIs have been the mainstay treatment for BCs, and other estrogen dependent cancers/disorders, in post-menopausal women. However, our data, others as well, demonstrate that aromatase is markedly, but non-discriminately, expressed in both human and mouse hormone-dependent and hormone-independent breast tumors, in addition to their non-cancerous counterparts [9,21,22,23]. Therefore, while the association of aromatase in breast physiology as well as pathophysiology is unquestionable, its expression levels do not differentiate cancer from normal tissue. Clearly, aromatase by itself is not the driver of ER+ BC. Concomitantly, we observed that varied expression of StAR is closely associated with E2 levels in both human and mouse cancerous and non-cancerous breast tissues. In line with the present data, amplification of the *StAR* gene, but neither aromatase nor other steroidogenic enzyme genes, as analyzed by TCGA (The Cancer Genomic Atlas) and METABRIC (Molecular Taxonomy of Breast Cancer International Consortium) BC datasets, correlates with poor survival of patients afflicted with luminal subtype BCs [7,9]. Moreover, expression of low and high StAR mRNA levels with two quartile combinations (<50% and >50%, <25% and >25%), in conjunction with the T (tumor) N (node) M (metastasis) staging, has been reported with TNM stage-dependent deaths of BC patients [7]. Hence, it is conceivable that StAR acts as a tumor promoter and/or oncogene in hormone-sensitive BCs.

**Figure 8:**
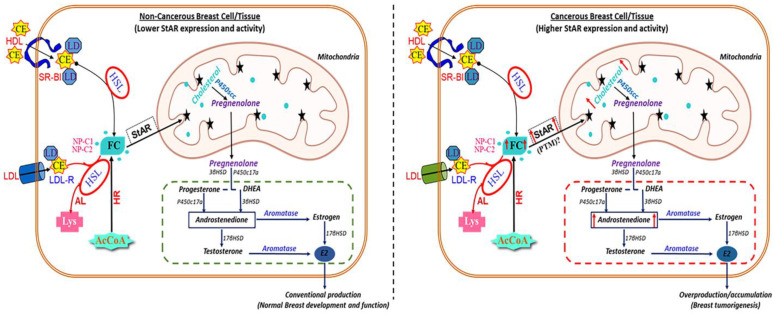
A schematic model illustrating the potential mechanism of StAR-mediated cholesterol utilization in non-cancerous and cancerous breast cells/tissues. Cholesterol used for steroidogenesis derives from a variety of sources. Circulating lipoproteins (high-density lipoprotein, HDL or low-density lipoprotein, LDL) bind to scavenger receptor, class B type 1 (SR-B1) and LDL-receptor, respectively, to release cholesterol esters (CEs) into cells. Hormone-sensitive lipase (HSL) generates free cholesterol (FC) from CEs. FC also arises from de novo synthesis from acetyl-CoA. The conversion of CEs into FC serves as an important step in controlling cholesterol availability for steroidogenesis. The StAR protein regulates steroid biosynthesis by controlling the intra-mitochondrial transport of cholesterol. At the mitochondria, the cytochrome P450scc enzyme converts cholesterol to pregnenolone, which exits the mitochondria and is then converted to various steroid hormones, including androgens and estrogen/E2, by various enzymes. Under physiologic conditions, StAR expression is moderate in breast cell/tissue, supporting appropriate synthesis of E2 for normal breast development and function (**left panel**). However, in cancerous breast cell/tissue, StAR expression is aberrantly high that presumably facilitates abnormal cholesterol delivery to the inner mitochondrial membrane and, as a consequence, abundant availability of androgen precursors for E2 accumulation in promoting tumorigenesis (**right panel**). Green and red dotted rectangles illustrate conventional and overproduction of E2 associated with normal breast development/function and breast tumorigenesis, respectively. LDL-R, LDL receptor; AL, acid lipase; Lys, lysosome; HR, HMG-CoA reductase; NP-C1/C2, Niemann-Pick type C1/C2; ↑, increase expression/activity.

A novel aspect of the present findings is the abundant expression of StAR, along with E2 accumulation, in three different Tg mouse models of spontaneous breast tumors driven by MMTV-Neu (HER+), MMTV-HRAS (ER+), and MMTV-PyMT (ER+/PR+). Conversely, both StAR and E2 levels were markedly lower in hormone-independent TNBC breast tumors and/or normal mammary tissue. Alternatively, aromatase is expressed abundantly in both tumorous and non-tumorous breast tissues, and its levels are extensively similar to human diseased and non-diseased breast cells, without any significant differences. These mouse lines are well-characterized and have been frequently used in studying a variety of BC functions [29,39,40,41]. Importantly, the results obtained with differential expression of StAR and corresponding changes in E2 levels in these cancerous and non-cancerous mouse breast tissues, reflected closely with human malignant and non-malignant breast cell lines. In accordance, preliminary data obtained with primary human breast tumor tissue cDNA array (BCRT101; Origene Technologies, Rockville, MD, USA) displayed relatively higher expression of *StAR* mRNA in ER+/PR+ BCs, when compared with either TNBC or normal mammary epithelial tissue (Manna, PR, unpublished observation). Note that the cDNA array data associated with breast samples (7-normal and 41-tumors) are of significantly mixed types with varied ages, tumor stages/grades, along with diverse pathological features. Among those 48 breast samples, careful analyses of data by Origene, identified 11-ER+/PR+, 7-TNBC, and 3-normal, warranting additional in-depth analysis for validation. Whereas StAR was found to be distinctly expressed in human and/or mouse hormone-sensitive breast tumor cells and tissues, an involvement of STARD3, a late endosomal membrane protein with ~37% C-terminal homology to StAR, cannot be excluded [42,43]. STARD3 was initially cloned as a gene amplified in BCs, and its overexpression has been demonstrated with increased cholesterol biosynthesis in HER2 + BCs [44,45]. Regardless of the regulatory mechanisms involved, it is tempting to speculate that aberrant high expression of StAR facilitates increased delivery of cholesterol to the inner mitochondrial membrane resulting in E2 accumulation for promoting breast tumorigenesis. This agrees well with relatively low expression of StAR that is required to synthesize estrogen/E2 necessary for the development/function of mammary tissue under normal conditions. These non-diseased and diseased states, associated with breast tissues, are influenced by a variety of factors and signaling. Based on these scenarios, a schematic model elucidating the function of StAR as a cholesterol transporter in mitochondria of mammary epithelial cells, under physiologic and pathophysiologic conditions, is proposed and illustrated in Figure 8.

The mechanism accounting for higher E2 accumulation in human and/or mouse hormone-sensitive BC cells/tissues are expected to be complex. Previously, we [46,47,48] and others [49,50] have demonstrated that post-translational modification (PTM) of StAR, connecting phosphorylation at Ser195/194 (human/mouse), by cAMP/PKA dependent mechanisms, enhances the maximum cholesterol transferring capability of StAR to the mitochondria for optimal steroid biosynthesis. While we reported that StAR was not endogenously phosphorylated in a variety of classical and non-classical steroidogenic tissues [47,48,51], a novel PTM of StAR, involving acetylation was uncovered, in which a total of 12 lysine residues undergoing acetylation/deacetylation were identified by liquid chromatography-tandem mass spectrometry [21]. These StAR lysine (K) residues were recognized under basal and HDACI-treated conditions, inferring they might have diverse effects on E2 biosynthesis [9,21]. Among these StAR acetylomes, point mutations of K111 and K253, acetylated frequently in ER+BC cells, to K111R (arginine) and K252R, deacetylation mimetics, resulted in suppression of E2 levels in MCF7 cells (Manna PR et al., unpublished observations and a manuscript in preparation), suggesting that StAR acetylation plays an important role in regulating E2 synthesis. In peri- and post-menopausal women, estrogen/E2 produced in either extra-ovarian sites or locally within breast tumors has been demonstrated to be key drivers of BCs via paracrine and/or intracrine mechanisms [9,52,53], suggesting that E2 derived from multiple sources promotes breast tumorigenesis.

An intriguing aspect of the present study is the inhibition of StAR expression and subsequent reduction in E2, by various HDACIs, in primary cultures of enriched breast tumor epithelial cells isolated from MMTV-PyMT mice. Breast tumors in this Tg mouse line are hormone-sensitive and display abundant expression of StAR, along with elevated E2 synthesis. Moreover, these mice possessed markedly higher E2 levels not only in breast tumors but also in circulation, resembling ER+/PR+ BCs in humans [12,18]. As such, MMTV-PyMT mouse line could be an appropriate preclinical animal model for studies of hormone-sensitive BCs. Importantly, a variety of HDACIs, at therapeutically relevant doses, were capable of suppressing StAR expression and E2 biosynthesis in hormone-responsive human MCF7 cells, findings in agreement with our previous observations [21]. Epigenetic modifiers are targeted in many therapeutic approaches for BCs, and our data provide pertinent evidence demonstrating that the efficacy of HDACIs in hormone-sensitive BC might involve repression of StAR expression with resultant decrease in E2 synthesis. This also raises the possibility that StAR itself could serve as a drug target for development of new line of small molecules to block the function of StAR for treatment of the most prevalent hormone-sensitive BCs.

## 4. Materials and Methods

### 4.1. Cell Lines, Animals, and Reagents

Hormone-sensitive cancerous MCF7 (HTB-22), MDA-MB-361 (HTB-27), and T-47D (HTB-133), and hormone-independent TNBC MDA-MB-468 (HTB-132), BT-549 (HTB-19), and MDA-MB-231 (HTB-26) cells, and non-cancerous HMEC (PCS-600-010), MCF10A (CRL-10317) and MCF12F (CRL-10783) breast cell lines were obtained from ATCC (Manassas, VA, USA), and were maintained in specific growth media containing antibiotics [11,21,22]. All cell lines were tested regularly for mycoplasma contamination and were utilized within 20 passages.

Three different transgenic (Tg) mouse (C57BL/6) models of spontaneous breast tumors, namely MMTV-Neu (Cat.# 002376), MMTV-HRAS (Cat.# 004363) and MMTV-PyMT (polyoma middle T antigen; Cat.# 002374), were obtained from the Jackson Laboratory (Bar Harbor, Maine, ME, USA). In addition, one TNBC mouse line (Cat.# 013591), expressing SV40 large T-antigen driven by rat prostatic steroid-binding protein C3(1) gene, was obtained from the Jackson Laboratory. Tg mice bearing breast tumors are categorized as the following: MMTV-Neu (HER2+), MMTV-HRAS (Ras activation-dependent), and MMTV-PyMT (ER+), in which tumors occur at ages 8–10 months in the first two lines, and at ages 2–3 months in the last line [29]. TNBC mouse develops tumors at 3–6-month ages. Normal mammary tissues from adult (similar genetic background) wild-type mice (~10 week) were used as controls. The breeding protocols (#15002) and experiments (#18005) with mice were approved by the Institutional Animal Care and Use Committee (IACUC) at the Texas Tech University Health Sciences [28,29]. Mice were sacrificed by cervical dislocation under CO_2_ anesthesia in accordance with the guidelines from the American Veterinary Medical Association.

Key reagents such as Panobinostat (LBH589), Entinostat (MS-275), PCI-34051 (A4091), and Anastrozole were purchased from APExBIO (Houston, TX, USA); and SAHA (Vorinostat), Inhibitor IV (Sirtuin 1 inhibitor), and Romidepsin (Istodax, FR228) were purchased from Millipore-Sigma (St. Louis, MO, USA). Different antibodies (Abs) used in the present study were obtained from the following sources: StAR (ab96637 or ab180804 or ab233427; Abcam, Boston, MA, USA), aromatase (ab124776; Abcam), and β-actin (Ambion, Austin, TX, USA).

### 4.2. Immunoblotting

Immunoblotting studies were performed using total cellular protein [5,21,48]. Briefly, various cancerous and non-cancerous breast cells and/or tissues were washed with 0.1 M PBS, homogenized in Pierce RIPA lysis buffer containing protease inhibitor cocktail (Thermo Fisher Scientific, Rockford, IL, USA), centrifuged at 12,000 g for 10 min, and supernatant was used for determining total protein [21,54]. Equal amounts of protein (50–70 μg) were loaded onto 10–12% SDS-PAGE (Bio-Rad Laboratories, Hercules, CA, USA). Upon electrophoresis, proteins were transferred onto methanol activated Immuno-Blot PVDF membranes, which were probed with specific Abs that recognize StAR (1:1000), aromatase (1:2000), and β-actin (1:10,000) [21]. Following overnight incubation with primary Abs, the membranes were washed and incubated with appropriate horseradish peroxidase-conjugated secondary Abs against rabbit or mouse IgG for 1 h at RT. The immunodetection of different proteins was determined using a SuperSignal West Femto Chemiluminescence kit (Thermo Fisher Scientific), and the intensity of different protein bands was quantified using an image analyzer (ImageJ or Quantity One Software) as described previously [5,21,48].

### 4.3. Immunofluorescence

Cancerous hormone-dependent (MCF7) and hormone-independent (TNBC; MB-231), and non-cancerous (MCF12F) breast cells were seeded in 6-well plates (3 × 10^5^ cells/well) containing coverslips (12 mm), 24 h prior to immunofluorescence staining [8,23]. Briefly, cells were washed with 0.1 M PBS and fixed with 4% paraformaldehyde for 15 min at RT, followed by quenching with 50 mM NH_4_Cl, and then permeabilized with 1% Triton X-100 for 10 min. Different cell types containing coverslips were blocked with 4% BSA for 30 min followed by 1 h incubation with StAR (1:200) and aromatase (1:300) Abs. After incubation, cells were washed with PBS, and incubated with Alexa flour 488 and 565 secondary Abs (both at 1:600; Thermo Fisher Scientific, Waltham, MA, USA) for 1 h at RT. Cells were then rinsed with PBS for 5 min and mounted with Pro-Long Gold antifade mounting solution with DAPI (Thermo Fisher Scientific), cured overnight at RT, and stored at −20 °C until immunofluorescence analysis. The images were captured using a laser scanning confocal microscope Nikon T-1E with a 60X objective and analyzed using NIS software [8,23].

### 4.4. Extraction of RNA and, Semi-Quantitative RT-PCR and Real-Time PCR

Total RNA was extracted from different human and mouse breast cells and/or tissues using either TRIzol reagent or PureLink RNA mini kit (Invitrogen, Carlsbad, CA, USA) [5,11,29,51]. Total RNA (1–1.5µg) was reverse transcribed using iScript cDNA synthesis kit (BioRad) or high-capacity cDNA reverse transcription kit (Invitrogen) after eliminating the genomic DNA. PCR primers were designed using either Primer-BLAST (NCBI) or PrimerBank (Table 1). At a minimum, 1/10th of synthesized cDNA was used in a final volume of 20µL in RT-PCR reaction, which contained 10µL of iTaq Universal probes supermix, and 20 pmol of respective primer/probe. PCR and qPCR were performed with either Takara Taq Hot Start Version (TaKaRa Biotechnology, Shiga, Japan) or Power SYBR Green PCR master mix (Bio-Rad). Expression of different genes (*StAR, CYP19A1, ERα (ESR1), ERβ (ERS2),* and *PR*) was normalized with the corresponding housekeeping β-actin gene and relative mRNA levels were calculated by the 2^−ΔΔ^Ct method [5,29,51]. Specific bands in semi-quantitative RT-PCR gel images were quantified as above.

### 4.5. Determination of E2 by Enzyme-Linked Immunosorbent Assay (ELISA)

Accumulation of E2 was determined using ELISA Kit obtained from Cayman Chemical Co. (Ann Arbor, MI, USA) [11,21]. Briefly, cell culture media and/or tissue homogenates, collected from different groups, were extracted with dichloromethane (4:1, *v/v*), snap frozen in ice/ethanol bath, and top solvent layer was poured into different tubes. The solvent containing E2 was dried at either air or in a speed vac, resuspended in ELISA assay buffer, and E2 levels were measured. The sensitivity of E2 assay was 15 pg/mL, and intra-assay coefficient of variation was below 10%. Assays were performed in duplicates and absorbance was read at 412 nm in a Microplate Reader [11,21].

### 4.6. Isolation and Primary Culture of Breast Tumor Epithelial Cells from MMTV-PyMT Mice and Treatment of These Cells with HDACIs

Isolation of breast tumor epithelial cells were performed under optimized conditions, following previously described procedures [55,56]. Briefly, MMTV-PyMT Tg mice possessing breast tumors (sizes 100–200 mm^3^) were sacrificed, carefully removed tumors, washed with 0.1 M PBS, and then placed on Petri dishes kept on ice. Upon washing, tumors were transferred to serum-free DMEM/F12 medium containing penicillin (100 IU/mL) and streptomycin (100 μg/mL). Breast tumors (2–4 from each mouse) were minced in digestion media (DMEM/F12 + 0.3% collagenase + 0.25% trypsin), poured into a 50 mL conical tube, and incubated for 4 h at 37 °C. Following incubation, digestion media were diluted with 0.2% BSA and the suspension was filtered through a sterile 40μM nylon mesh. The filtered cells were centrifuged at 300 g for 5 min and supernatant was aspirated out carefully. Cells were then resuspended in DMEM/F12 + 0.2% BSA + 0.25% trypsin media, incubated for 10–15 min, and centrifuged at 300 g for 5 min to discard the supernatant. Red blood cells in cell pellets were lysed with NH_4_Cl/PBS and 1% BSA (4:1) for 4 min and cell mixture was then centrifuged at 300 g for 5 min. Upon removing supernatant, the pellet was washed twice with DMEM/F12 containing 0.2% BSA. To remove fibroblasts, cells were transferred to T-175 cm^2^ flask and cultured in an incubator for 1 h. Epithelial cells were carefully removed from fibroblasts attached to the flask. Breast tumor enriched epithelial cells were cultured in an incubator for 72 h before using them for treatments [55,56].

### 4.7. Statistical Analysis

All experiments were conducted in a randomized and blinded manner, and experiments were repeated at least three times, as specified in figure legends. Statistical analyses were performed by one-way analysis of variance (ANOVA) followed by Fisher’s protected least significant difference test using Statview (Abacus Concepts, Inc., Berkeley, CA, USA). In addition, Student’s T-tests were used for analyzing significant differences between two groups using GraphPad Prism (GraphPad Software, La Jolla, CA, USA). These tests were used to determine whether the differences observed in various experiments among diverse groups were statistically significant. Data presented are the mean ±SE, and results are considered significant at *, *p* < 0.05; **, *p* < 0.01; ***, *p* < 0.001, and ****, *p* < 0.0001.

## 5. Conclusions

Differential expression of StAR, but not aromatase, and its functional relevance to E2 synthesis in human and mouse cancerous and non-cancerous breast cells/tissues, elucidating the potential for this cholesterol transporter as a diagnostic/prognostic marker, was demonstrated using three independent approaches. First, a number of hormone-dependent, but not hormone-independent, human BC cell lines were found to overexpress StAR, with little to none in their normal counterparts, in which StAR expression correlated with E2 biosynthesis. Second, primary human breast tumor tissue cDNA array showed higher expression of *StAR* mRNA in ER+/PR+ subtypes in comparison to its modest levels in TNBCs and normal mammary epithelial tissue. Third, three different Tg mouse models of spontaneous breast tumors documented aberrantly high expression of StAR, along with E2 accumulation, in hormone-sensitive breast tumors, over the levels seen in either TNBCs or normal mammary tissue. These findings point to the critical influence of StAR in the biologic behavior and/or pathogenesis of hormone-sensitive BCs. Noteworthy, inhibition of StAR expression, corresponding with a reduction in E2 biosynthesis, by a variety of HDACIs, in both hormone-sensitive human MCF7 cells and mouse enriched breast tumor epithelial cells, demonstrated that StAR could be targeted for combating ER+ BC. Based on the above considerations, we conclude that StAR could serve as a novel diagnostic marker selective for ER+/PR+ BC subtypes and that pharmacological targeting of this protein, either blockage of its function or suppression of its expression, has the potential for treatment of hormone-sensitive BC.

## Figures and Tables

**Table 1:** Primers used for RT-PCR and/or RT-qPCR for analyzing expression of different genes.

Genes	Forward Primer	Reverse Primer
Human StAR	CTG GGA GCT CCT ACA GAC AC	AGC CGA GAA CCG AGT AGA GAG
Mouse StAR	#1: GGA GCT CTC TGC TTG GTT CTC, or #2: GAC CTT GAA AGG CTC AGG AAG AAC	#1: TTA GCA CTT CGT CCC CGT TC, or #2: TAG CTG AAG ATG GAC AGA CTT GC
Mouse ERα (ESR1)	ATT ATG GGG TCT GGT CCT GC	CTT TCC GTA TGC CGC CTT TC
Mouse ERβ (ESR1)	AGA CGA AGA GTG CTG TCC CA	GGG GTA CAT ACT GGA GTT GAG G
Mouse PR (PR)	ATC TGG CTG TCA CTA TGG CG	ACT TAC GAC CTC CAA GGA CCA
Human CYP19A1	GGC AGT GCC TGC AAC TAC TA	GTC ACC TCC TCC AAC CTG TC
Mouse CYP19A1	AAC ATG CTC TTC CTG GGG AT	GTC CTT GAC GGA TCG TTC ATA C
Human β-actin	GGA CTT CGA GCA AGA GAT GG	AGC ACT GTG TTG GCG TAC AG
Mouse β-actin	CTG GCA CCA CAC CTT CTA	GGG CAC AGT GTG GGT GAC

## Data Availability

Data in relation to the studies reported here are provided in this manuscript. In addition, relevant protocols and other information can be obtained from the corresponding author upon reasonable request.

## Abbreviations

ATCC	American type culture collection
BC	breast cancer
StAR	steroidogenic acute regulatory protein
E2	17β-estradiol
ER+/PR+	estrogen progesterone receptor positive
TNBC	triple negative BC
HDACIs	histone deacetylase inhibitors
PANO	Panobinostat
ROMI	Romidepsin
ENTI	Entinostat
PCI	PCI-34051
IV	inhibitor IV
ELISA	enzyme-linked immunosorbent assay
MMTV	mouse mammary tumor virous
PyMT	polyoma middle T antigen
